# Safety monitoring of Imvamune vaccine during the 2022 mpox outbreak in Canada

**DOI:** 10.14745/ccdr.v51i08a05

**Published:** 2025-08-28

**Authors:** Charlotte Wells, Yuhui Xu, Ashley Weeks, Amanda Shaw, Susanna Ogunnaike-Cooke

**Affiliations:** 1 Public Health Agency of Canada

**Keywords:** mpox, adverse events, Imvamune, vaccine safety, outbreak

## Abstract

**Background:**

In Canada in 2020, the indication for use of Imvamune was expanded to include immunization against smallpox, mpox and related *Orthopoxvirus* infection and disease in adults who are 18 years of age and older and determined to be at high risk for exposure.

**Methods:**

Since the introduction of this new use for the vaccine and throughout the 2022 mpox outbreaks, the Public Health Agency of Canada (PHAC) has closely monitored the safety of the Imvamune vaccine through the Canadian Adverse Events Following Immunization Surveillance System (CAEFISS).

**Results:**

This article describes reports of adverse events following immunization (AEFI) after administration of Imvamune, submitted to the CAEFISS database between May 24, 2022 and December 11, 2022, during the activation of Canada’s emergency response.

**Conclusion:**

Monitoring of AEFI reports following immunization with Imvamune submitted to CAEFISS has not identified any new or unexpected safety concerns in the Canadian adult population. The Public Health Agency of Canada continues to monitor for potential vaccine safety signals.

## Introduction

### Issue identification

In May 2022, human mpox cases were reported in the United Kingdom and other countries shortly before being identified in Canada ((1,2)). In July 2022, the World Health Organization declared the mpox outbreak a public health emergency of international concern (3). By December, more than 80,000 cases of mpox were reported across 110 countries ((4)). The severity of reported cases within this outbreak was low, with few hospitalizations and no deaths; however, cases reported considerable pain from lesions or scarring ((5–7)). Cases occurred predominantly within marginalized communities, specifically gay, bisexual and other men who have sex with men (GBMSM) ((5)). The term GBMSM aims to include people who self-identify as cisgender or transgender men whose sexual partners are cisgender and/or transgender men, regardless of their sex assigned at birth; that being said, there may be differences across surveillance systems with how gender identity, sex, and sexual orientation is defined ((5)). Canadian provinces and territories initiated an immunization campaign ((8)) following the release of interim guidance by the National Advisory Committee on Immunization (NACI) on the use of Imvamune in the context of monkeypox outbreaks in Canada ((8,9)). NACI further indicated that additional post-marketing safety data on Imvamune was a research priority ((9)).

Imvamune (modified vaccinia Ankara-Bavarian Nordic), also known outside of Canada by the brand names Jynneos and Imvanex, is a live-attenuated, non-replicating *Orthopoxvirus* vaccine ((10)). Health Canada initially granted approval of Imvamune in 2013 under the Extraordinary Use New Drugs (EUNDs) submission for use against smallpox infections ((11)). A supplement to the EUNDs in 2020 expanded the indication to include mpox and related orthopoxviral infection ((11)). The approval was for primary doses of the vaccine administered as two 0.5 mL doses given subcutaneously, at least four weeks apart. Imvamune can be given prior to potential exposure or as post-exposure prophylaxis for individuals who have been exposed to the virus but do not yet have symptoms of mpox ((9)).

The Public Health Agency of Canada (PHAC) monitored Imvamune vaccine safety using the Canadian Adverse Events Following Immunization Surveillance System (CAEFISS), which is a federal, provincial, and territorial public health post-market vaccine safety surveillance system coordinated by PHAC and used to continuously monitor the safety of vaccines administered in Canada ((12)). Reports are first submitted to a health authority (i.e., local, regional, provincial/territorial or federal authorities) by healthcare providers of individuals who experienced an adverse event following immunization (AEFI). These reports are then submitted by the federal, provincial or territorial health authorities to PHAC for inclusion in the CAEFISS database ((12)). Adverse events following immunization are also monitored through the Canada Vigilance Program of Health Canada, however, no reports of AEFIs with Imvamune were received through this program.

The aim of this study was to describe AEFI reports following administration of Imvamune submitted to the CAEFISS database between May 24, 2022 and December 11, 2022. This study period was defined due to the peak of the outbreaks that occurred in Canada during this year ((2,5)) and the national health portfolio emergency response being de-escalated in December 2022 ((13)).

## Methods

Data were searched and extracted data from CAEFISS for deidentified AEFI reports submitted between May 24, 2022 and December 11, 2022. Data extraction was done using the “Preferred Terms” hierarchical level of the Medical Dictionary for Regulatory Activities (MedDRA) standardized terminology ((14)). All reports underwent systematic primary medical case review by trained health professionals. An AEFI was considered serious if it resulted in death, was life-threatening (an event/reaction in which the patient was at real, rather than hypothetical, risk of death at the time of the event/reaction), required in-patient hospitalization or prolongation of existing hospitalization, resulted in persistent or significant disability/incapacity, or resulted in a congenital anomaly/birth defect ((15)). Dose-administered data were also collected from relevant provincial and territorial partners.

Reporting rates of adverse events were calculated by dividing the number of adverse events by the number of doses administered. The associated 95% confidence intervals (CIs) were estimated using the Poisson exact method. The data extraction and analysis for this article were generated using R Statistical Software version 4.2.2 ((16)) and SAS Enterprise Guide software version 7.1. The SAS and all other SAS Institute Inc. product or service names are registered trademarks or trademarks of SAS Institute Inc., Cary, North Carolina, United States.

## Results

Between May 24, 2022 and December 11, 2022, 119,826 doses of Imvamune were administered in Canada, including 95,346 (79.6%) first doses, 24,478 (20.4%) second doses, and two third doses (0.0%). The majority (93.2%) of doses were administered to males. Imvamune was administered to 80 persons aged less than 18 years.

A total of 53 AEFI reports following administration of Imvamune were submitted to PHAC for inclusion in the CAEFISS database during the period under analysis, corresponding to an overall reporting rate of 44.2 reports per 100,000 doses (95% CI: 33.1–57.9). Most reports were classified as non-serious based on the described clinical manifestations and outcomes, with AEFIs reported predominantly among males (81%), primarily in the age range of 0 to 49 years (72%) (**Table 1**). This is consistent with the recommendations for Imvamune use in the context of an active mpox outbreak, as well as the epidemiology of the 2022 outbreak ((5)). While most reports indicated vaccination via subcutaneous injection (53%), route of administration was not provided for many reports (Table 1). The most reported adverse events were similar between vaccinations administered subcutaneously and those where route of administration was unknown (**Table 2**).

**Table 1:** Characteristics of Imvamune vaccine recipients with adverse events following immunization reports submitted to CAEFISS in Canada, May 24, 2022–December 11, 2022, (n=53)

Characteristic	Number of reports (%)^a^
**Sex**	
Male	43 (81%)
Female	6–9
Other	<5
**Age group (in years)**	
0–29	12 (23%)
30–39	14 (26%)
40–49	12 (23%)
50–59	10 (19%)
60+	5 (9%)
**Route of administration**	
Subcutaneous	28 (53%)
Intramuscular^b^	<5
Unknown	21–24
**Seriousness**	
Non-serious	49–52
Serious	<5

Abbreviation: CAEFISS, Canadian Adverse Events Following Immunization Surveillance System

^a^ For cells with small counts (n<5), the exact number and proportion is supressed due to potential personal identifiers

^b^ This route was indicated as an immunization error

**Table 2:** Top 10 most frequently reported adverse events following immunization after Imvamune vaccine administration, by route of vaccine administration from CAEFISS in Canada, May 24, 2022–December 11, 2022

AEFI^a^	Number of reports^b^
**Subcutaneous route of administration**	
Vaccination site erythema	9 (32%)
Vaccination site pain	9 (32%)
Vaccination site nodule	6 (21%)
Vaccination site swelling	6 (21%)
Vaccination site warmth	6 (21%)
Erythema	<5
Pruritus	<5
Urticaria	<5
Vaccination site induration	<5
Rash	<5
**Unknown route of administration**	
Vaccination site pain	13 (54%)
Vaccination site erythema	10 (42%)
Vaccination site swelling	8 (33%)
Vaccination site mass	5 (21%)
Pruritus	<5
Rash	<5
Vaccination site nodule	<5
Vaccination site pruritus	<5
Erythema	<5
Vaccination site cellulitis	<5

Abbreviations: AEFI, adverse event following immunization; CAEFISS, Canadian Adverse Events Following Immunization Surveillance System

^a^ Note that each report represents one person and may contain information on more than one AEFI

^b^ For cells with small counts (n<5), the exact number and proportion is supressed due to potential personal identifiers. Proportions are calculated using total number of reports for each route of administration

The majority of AEFIs reported were local reactions associated with the vaccination site. Notably, vaccine site pain was the most reported, with 18.4 reports per 100,000 doses administered (**Table 3**). There were no reports of anaphylaxis.

**Table 3:** Reporting rates per 100,000 doses administered for the 10 most frequently reported adverse events following immunization after Imvamune vaccine administration from CAEFISS in Canada, May 24, 2022–December 11, 2022

AEFI^a^	Number of events	Reporting rate (95% CI)
Vaccination site pain	22	18.4 (11.5–27.8)
Vaccination site erythema	20	16.7 (10.2–25.8)
Vaccination site swelling	15	12.5 (7.0–20.6)
Vaccination site mass	9	7.5 (3.4–14.3)
Vaccination site nodule	9	7.5 (3.4–14.3)
Pruritus	8	6.7 (2.9–13.2)
Vaccination site warmth	8	6.7 (2.9–13.2)
Erythema	6	5.0 (1.8–10.9)
Rash	6	5.0 (1.8–10.9)
Vaccination site cellulitis	5	4.2 (1.4–9.7)

Abbreviations: AEFI, adverse event following immunization; CI, confidence interval; CAEFISS, Canadian Adverse Events Following Immunization Surveillance System

^a^ Note that each report represents one person and may contain information on more than one AEFI

All serious reports required hospitalizations and no deaths were reported. Medical case review did not confirm an association between the AEFIs reported and the vaccination or deemed this association as unclassifiable.

Serious AEFI reports included events such as cerebrovascular events, superficial vein thrombosis, and injection site reactions.

## Discussion

As of this report, monitoring of AEFIs following Imvamune administration has not identified any new or unexpected safety concerns in the Canadian adult population. The predominance of vaccine site reactions following injection aligns closely with what has been observed in clinical studies and with expected reactions listed on the product monograph ((10)). Furthermore, reporting rates of the most reported AEFIs are comparable to those reported in the United States as part of the mpox outbreak vaccination campaign ((17)).

Over the seven-month study period, national spontaneously reported data do not suggest any unexpected concerns regarding the number and nature of serious adverse event reports. With respect to other events of special interest, such as myocarditis, there were no reports of such events in Canada. However, given myocarditis was observed in the United States following vaccination with Jynneos at a rate of 1.53 and 2.99 per million doses after dose 1 and dose 2, respectively ((17)), it is unlikely that such rare adverse events would be observed given the limited use of this vaccine in Canada.

During the Imvamune vaccination campaign, NACI recommended a dose sparing strategy of intradermal injection for immunocompetent adults when given as a second dose, instead of using the standard subcutaneous injection method ((11)). Based on the data available in CAEFISS, there is no evidence to conclude that the route of vaccine administration may have had a notable impact on the rate of AEFI occurrence. Indeed, the top 10 commonly reported adverse events were similar between vaccinations administered subcutaneously and those where the route of vaccine administration was unknown. Furthermore, reporting from the United States suggests that the adverse events reported were not different between the two routes of administration ((17)).

### Limitations

The results of this investigation are subject to limitations of the CAEFISS reporting system, which include the potential for under-reporting, missing information in submitted reports (which, at times, led to an inability to confirm the diagnosis as reported), and different reporting practices between reporting jurisdictions. In addition, data on the number of doses that may have been administered to the recipient, as well as on the route by which the vaccine was administered (intradermal vs subcutaneous) were missing in a significant number of AEFI reports. This limited our ability to conduct route-specific rate calculations and relative risk comparisons. Finally, the occurrence of an AEFI report does not necessarily confirm that the AEFI meets standard diagnostic criteria or that a causal link exists between the administration of a vaccine and the occurrence of the reported adverse event.

## Conclusion

In conclusion, as of this report, monitoring of AEFIs following Imvamune did not reveal any unexpected safety concerns in the Canadian adult population. The adverse events observed during the analysis period align well with published data and serious events were rare. Post-marketing Imvamune safety surveillance studies are limited, and this article adds to the knowledge base of Imvamune outside of clinical trials. The Public Health Agency of Canada will continue to monitor AEFI reports as they are submitted to the CAEFISS reporting system.
